# Near infrared II excitation nanoplatform for photothermal/chemodynamic/antibiotic synergistic therapy combating bacterial biofilm infections

**DOI:** 10.1186/s12951-023-02212-7

**Published:** 2023-11-24

**Authors:** Xuanzong Wang, Chi Zhang, Liuliang He, Mingfei Li, Pengfei Chen, Wan Yang, Pengfei Sun, Daifeng Li, Yi Zhang

**Affiliations:** 1 Department of Orthopedics, The First Affiliated Hospital of Zhengzhou University, Zhengzhou, 450052 China; 2 State Key Laboratory of Organic Electronics and Information Displays & Institute of Advanced Materials (IAM), Jiangsu Key Laboratory for Biosensors, Nanjing University of Posts & Telecommunications, Nanjing, 210023 China

**Keywords:** Bacterial biofilm infection, pH-responsive nanoplatform, NIR-II photothermal therapy, Chemodynamic therapy, NIR-II light excitation

## Abstract

Drug-resistant bacterial biofilm infections (BBIs) are refractory to elimination. Near-infrared-II photothermal therapy (NIR-II PTT) and chemodynamic therapy (CDT) are emerging antibiofilm approaches because of the heavy damage they inflict upon bacterial membrane structures and minimal drug-resistance. Hence, synergistic NIR-II PTT and CDT hold great promise for enhancing the therapeutic efficacy of BBIs. Herein, we propose a biofilm microenvironment (BME)-responsive nanoplatform, BTFB@Fe@Van, for use in the synergistic NIR-II PTT/CDT/antibiotic treatment of BBIs. BTFB@Fe@Van was prepared through the self-assembly of phenylboronic acid (PBA)-modified small-molecule BTFB, vancomycin, and the CDT catalyst Fe^2+^ ions in DSPE-PEG_2000_. Vancomycin was conjugated with BTFB through a pH-sensitive PBA-diol interaction, while the Fe^2+^ ions were bonded to the sulfur and nitrogen atoms of BTFB. The PBA-diol bonds decomposed in the acidic BME, simultaneously freeing the vancomycin and Fe^2+^ irons. Subsequently, the catalytic product hydroxyl radical was generated by the Fe^2+^ ions in the oxidative BME overexpressed with H_2_O_2_. Moreover, under 1064 nm laser, BTFB@Fe@Van exhibited outstanding hyperthermia and accelerated the release rate of vancomycin and the efficacy of CDT. Furthermore, the BTFB@Fe@Van nanoplatform enabled the precise NIR-II imaging of the infected sites. Both in-vitro and in-vivo experiments demonstrated that BTFB@Fe@Van possesses a synergistic NIR-II PTT/CDT/antibiotic mechanism against BBIs.

**Supplementary Information:**

The online version contains supplementary material available at 10.1186/s12951-023-02212-7.

## Introduction

Bacterial biofilm infections (BBIs) pose a serious threat to public health [1, 2]. BBIs, in particular, are strongly resistant to both innate and adaptive host immune defenses, leading to long-lasting drug resistance [3, 4]. However, the inability of routine antibiotic treatments to penetrate or eradicate biofilms greatly decreases the therapeutic efficiency of the antibiotics and worsens the drug-resistance of the bacteria [5, 6]. Hence, the development of novel antibiofilm strategies to address the concerns associated with BBIs and drug resistance in patients has become imperative. Nanomedicine has broad prospects for treating BBIs [7, 8], particularly for enhancing the efficacy of antibiotics through various strategies, such as thermotherapy and reactive oxygen species (ROS) therapeutics. Both thermotherapy and ROS therapeutics can effectively disrupt the integrity of biofilm structures and simultaneously kill bacteria through efficient thermal conversion and strong oxidation [9–11]. In addition, these treatments rarely lead to antibiotic resistance. Thus, these treatments would have great therapeutic potential in the future.

Recently, photothermal therapy (PTT) with near infrared (NIR) absorption has been significantly improved [12–15]. Owing to its deep penetration, high allowable exposure, and noninvasiveness, near infrared II (NIR-II, 1000–1700 nm) photothermal therapy (NIR-II PTT) has been employed as a therapeutic strategy for treating bacterial infections [16, 17]. In addition, several special NIR-II absorption PTT agents enable NIR-II fluorescence imaging (NIR-II FI) [18, 19], and thus NIR-II FI-guided NIR-II PTT can integrate the diagnosis and treatment in all-in-one phototheranostics. Organic donor-acceptor-donor (D-A-D)-structured small molecules have several advantages over other NIR-II absorption materials because of their strong intrinsic absorption and extinction properties, high photothermal conversion ability, large Stokes shifts, and good NIR-II fluorescence [12, 13]. Moreover, D-A-D small molecules can be modified with functional groups that can conjugate drug molecules or irons, which will lead to satisfactory loading of antibiotics and their controllable release, depending on the pathological characteristics of the associated biofilm microenvironments (BMEs), such as those with acidic pH. These properties of organic D-A-D-structured small molecules highlight the great potential they have in further antibiofilm applications.

Owing to their strong oxidizing properties, ROS can fight bacteria and biofilms by increasing intracellular oxidation, denaturing proteins, oxidizing lipids, and damaging bacterial biofilm membranes and matrix structures [20–23]. Typically, ROS is produced by photodynamic therapy (PDT) [24–26], sonodynamic therapy (SDT) [27, 28] and chemodynamic therapy (CDT) [29–31]. However, PDT and SDT mainly rely on endogenous oxygen concentrations and possess limited in vivo efficacy and penetration. CDT utilizes endogenously overexpressed hydrogen peroxide (H_2_O_2_) in the lesion microenvironment and generate toxic hydroxyl radicals (·OHs) through a metal-catalyzed Fenton reaction [32, 33]. Compared with other ROS therapies, CDT is less dependent on external stimulation, capable of deep treatment, and significantly less toxic to the surrounding normal tissues [32, 33].

**Scheme 1:**
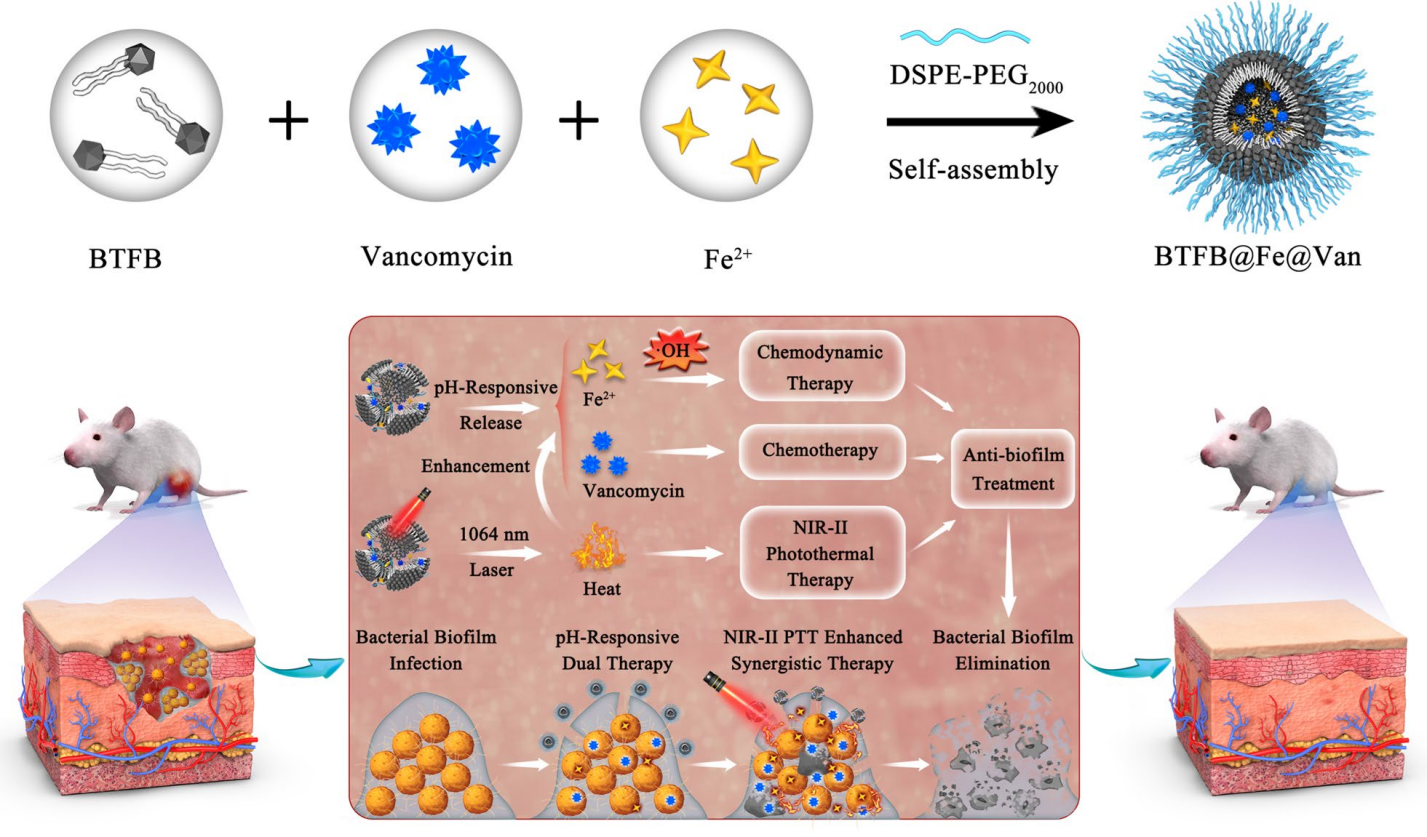
Schematic illustration of a BME-responsive nanoplatform BTFB@Fe@Van and its synergistic treatment for eradicating drug-resistant BBIs

In this study, a novel biofilm microenvironment (BME)-responsive nanoplatform, BTFB@Fe@Van, was developed to combat BBIs using NIR-II FI-guided synergistic NIR-II PTT/CDT/antibiotics mechanism. The BTFB@Fe@Van nanoplatform was prepared by co-encapsulating phenylboronic acid (PBA)-modified NIR-II organic small-molecule BTFB, the antimicrobial drug vancomycin (Van), and the CDT catalyst Fe^2+^ ions into DSPE-PEG_2000_ (Scheme 1). Van and Fe^2+^ ions were conjugated to BTFB within the micelle core via boronate ester bonds, chelation interactions with the PBA group, and backbone sulfur and nitrogen atoms. The acidic and oxidative BME would decompose the boronate ester bonds, promote the release of Van and Fe^2+^ ions, and generate cytotoxic ·OH through a Fenton reaction to initiate CDT. Importantly, under 1064 nm laser irradiation, BTFB@Fe@Van exhibited outstanding hyperthermia and accelerated the release rate of Van and efficacy of the Fenton reaction. The in vitro experiments showed that BTFB@Fe@Van possessed a noteworthy NIR-II PTT/CDT/antibiotic synergistic mechanism against BBIs. Moreover, BTFB@Fe@Van significantly promoted the recovery of *Staphylococcus aureus* (*S. aureus*) biofilm-infected tissues and reduced the inflammatory responses in vivo. Owing to the biological superiority of this trimodal synergistic strategy, BTFB@Fe@Van is an intelligent nanoplatform with great potential for eradicating drug-resistant BBIs.

## Results and discussion

### Synthesis and characterization of BTFB@Fe@Van

**Fig. 1:**
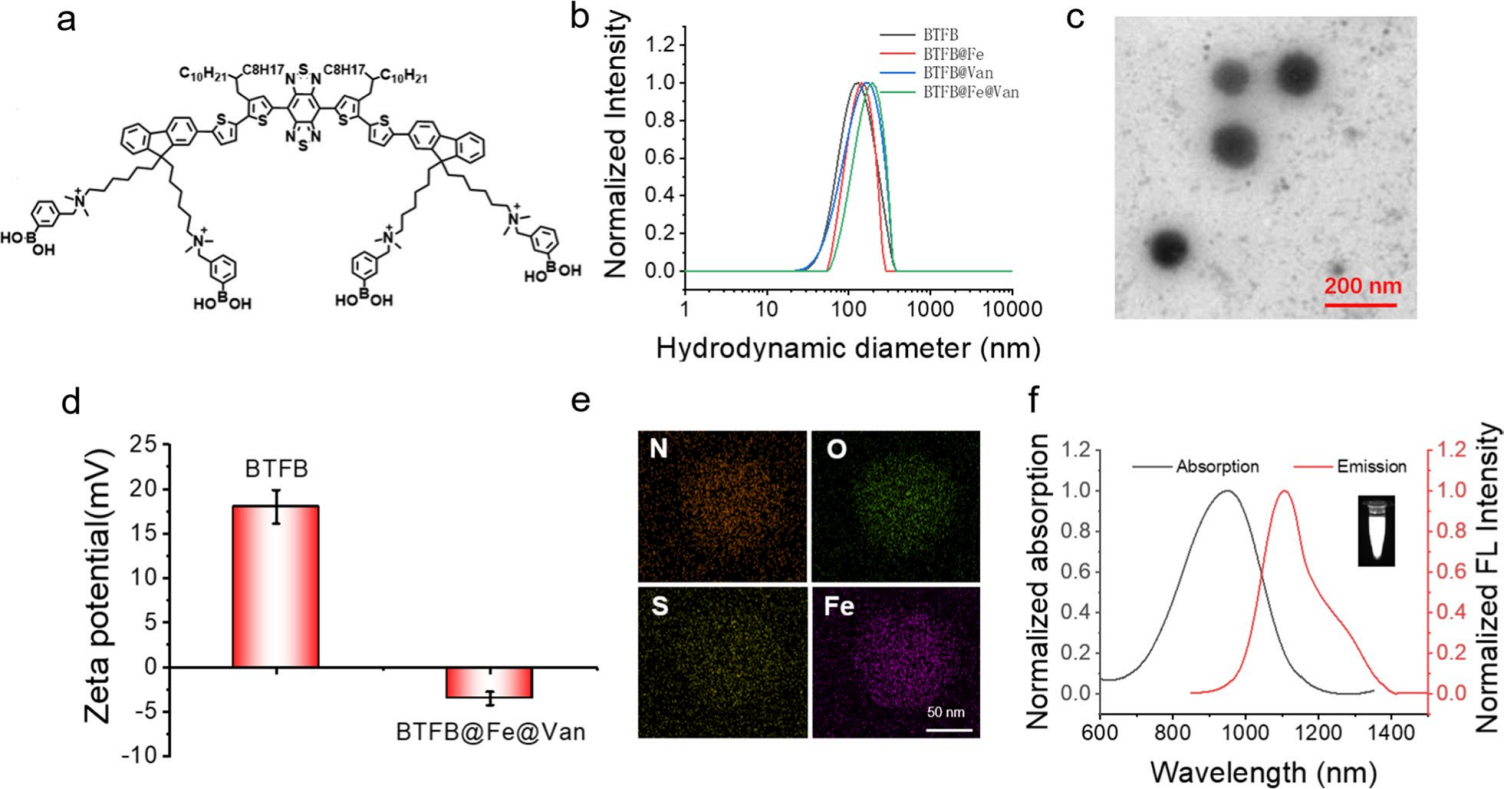
**a** Structural formula of NIR-II organic small molecule BTF-PBA. **b** Average hydrodynamic diameters of the synthesized BTFB, BTFB@Fe, BTFB@Van, and BTFB@Fe@Van using DLS. **c** TEM image of BTFB@Fe@Van. **d** Zeta potentials of BTFB and BTFB@Fe@Van in water. **e** Energy-dispersive spectroscopy elemental mapping diagram of BTFB@Fe@Van. Scale bar: 50 nm. **f** Absorption and NIR-II fluorescence spectrum of BTFB@Fe@Van under 808 nm laser excitation. (Inset: NIR-II images of BTFB@Fe@Van under 808 nm laser excitation with a 1064 nm filter)

The procedure used for synthesizing the multifunctional nanoplatform (BTFB@Fe@Van) is illustrated in Scheme 1. First, the NIR-II organic small molecule BTF-PBA was synthesized based on our recent work [34]. The BTFB@Fe@Van nanoparticles were prepared through the self-assembly of phenylboronic acid-modified NIR-II organic molecule BTF-PBA (Fig. 1a), antimicrobial drug Van, and Fe^2+^ ions. The dimethyl sulfoxide (DMSO) solution of BTF-PBA and Van was firstly mixed with the DSPE-PEG_2000_ water solution because of the dynamic boronate ester bonds between the PBA groups in BTF-PBA and the vicinal diols of Van. Subsequently, the CDT catalyst Fe^2+^ ions were added to the solution to obtain a green solution. This successful binding of Fe^2+^ ions was due to the abundant sulfur and nitrogen atoms in BTF-PBA. Meanwhile, the water-soluble nanoparticles packaged with only BTF-PBA (BTFB) or BTF-PBA and Fe^2+^ ions (BTFB@Fe) acted as the controls.

As shown in Fig. 1b, dynamic light scattering (DLS) results indicate that the average hydrodynamic diameters (*D*_h_) of BTFB, BTFB@Fe, BTFB@Van, and BTFB@Fe@Van were approximately 123, 134, 138, and 168 nm, respectively. The slightly higher *D*_h_ of BTFB@Fe@Van than that of BTFB was due to the encapsulation of the antimicrobial drug Van and Fe^2+^ ions. All four nanoparticles exhibited a uniform spherical morphology, as observed in the transmission electron microscopy (TEM) images (Fig. 1c and Additional file 1: Fig. S1). The *D*_h_ of BTFB@Fe@Van was also detected in a phosphate-buffered saline (PBS) solution containing 10% fetal bovine serum and Dulbecco’s Modified Eagle Medium. BTFB@Fe@Van had similar *D*_h_ values in different solutions (Additional file 1: Fig. S2). Even after BTFB@Fe@Van has been stored for 4 weeks, its particle size remained unchanged, suggesting its good dispersion stability (Additional file 1: Fig. S2). The Zeta potentials of the BTFB nanoparticles in the solution and BTFB@Fe@Van were measured. BTFB@Fe@Van had a negative surface charge (− 4.8 mV) (Fig. 1d). Energy-dispersive spectroscopy elemental mapping confirmed that C, O, and Fe were uniformly distributed in BTFB@Fe@Van (Fig. 1e).

The ultraviolet–visible–near-infrared (UV–VIS–NIR) absorption spectra shown in Fig. 1f revealed that the synthesized BTFB@Fe@Van had a broad absorption range of 700–1200 nm and that its extinction coefficient at 1064 nm was high at 3.19 L/g cm (Additional file 1: Fig. S3), which is consistent with the findings of our previous study [34]. The characteristic absorption of Van at 280 nm can also be observed in Additional file 1: Fig. S4, indicating that BTFB@Fe@Van had successfully entrapped Van. The Van-loading capacity of the nanoparticles obtained by calculation was 28%. Figure 1f also indicates that BTFB@Fe@Van has a strong NIR-II emission from 900 to 1300 nm upon excitation at 808 nm.

**Fig. 2:**
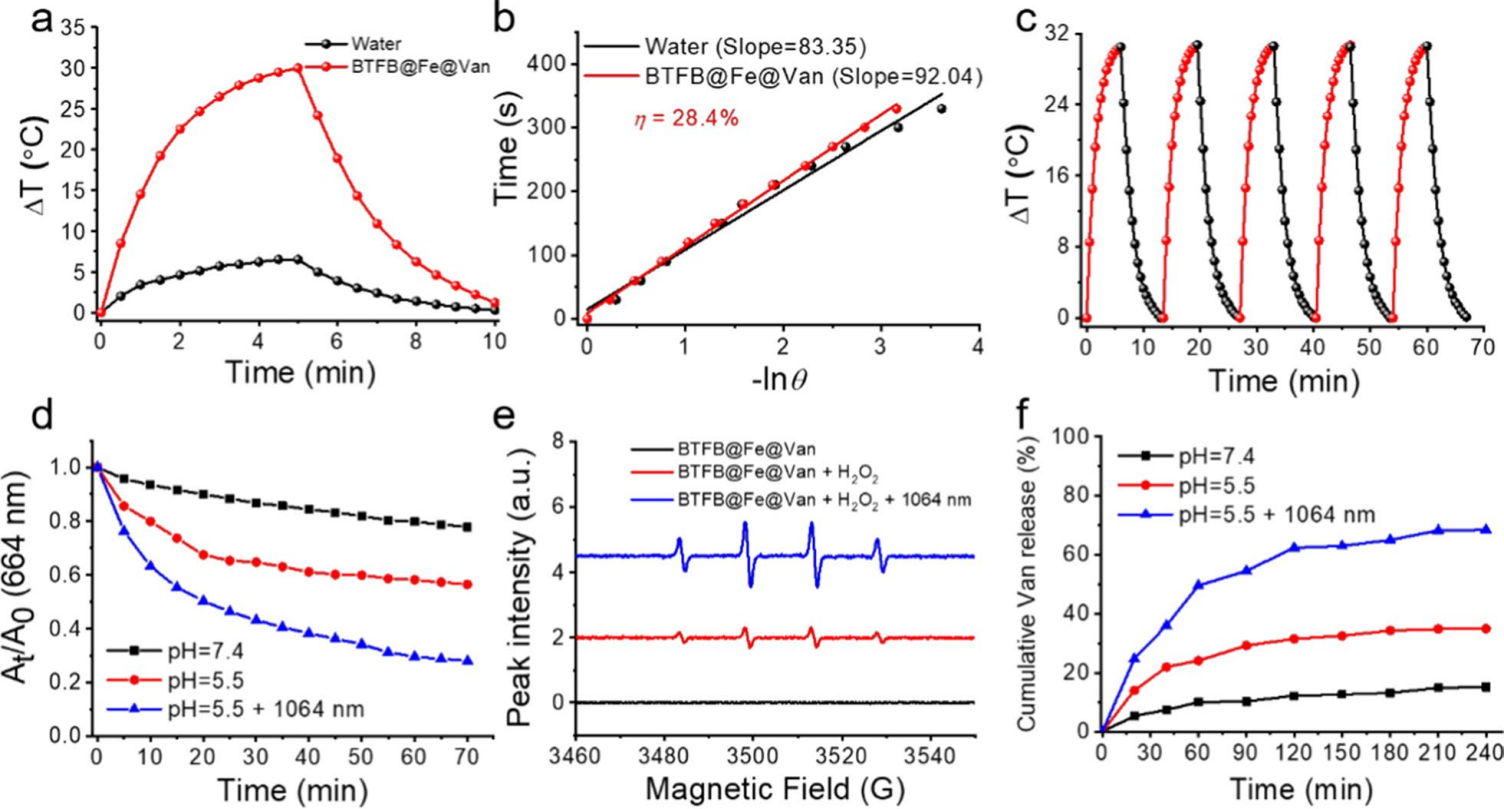
**a** Photothermal heating curves of water and BTFB@Fe@Van (0.1 mg mL^−1^) under 1064 nm laser irradiation (1.0 W cm^−2^). **b** Linear correlation between the cooling time and negative natural logarithm of the driving force temperature. **c** Photothermal stability of BTFB@Fe@Van over five on/off cycles. **d** ·OH generation quantified from the decrease in MB absorbance at 664 nm ([BTFB@Fe@Van] = 0.1 mg mL^−1^, [H_2_O_2_] = 0.1 mM, and [MB] = 1.0 mM) with different treatments [pH 7.4, pH 5.5, and pH 5.5 with 1064 nm laser irradiation (1.0 W cm^−2^)]. **e** ESR spectra of the aqueous solutions of BTFB@Fe@Van under different conditions ([H_2_O_2_] = 0.1 mM, and [H_2_O_2_] = 0.1 mM with 1064 nm laser irradiation (1.0 W cm^−2^)). **f** Van release from BTFB@Fe@Van under different environments (pH 7.4, pH 5.5, and pH 5.5 with 1064 nm laser irradiation (1.0 W cm^−2^)

The NIR-II photothermal performance of BTFB@Fe@Van was carefully explored considering its strong absorption at 1064 nm. As shown in Fig. 2a, the temperature of BTFB@Fe@Van increased by 30 °C after its laser irradiation at 1064 nm (1.0 W cm^−2^) for 5 min at a concentration of 0.1 mg mL^−1^, which was much higher than that of pure water whose temperature increased only by 7 °C under the same irradiation. The temperature rapidly increased with increasing BTFB@Fe@Van concentration (Additional file 1: Fig. S5). The photothermal conversion efficiency of BTFB@Fe@Van obtained by calculation was 28.4%, which is higher than the photothermal conversion efficiencies of most traditional photothermal agents (Fig. 2b). The photothermal performance of BTFB@Fe@Van exhibited no significant changes after five continuous cycles of laser on/off irradiation, indicating its high photostability (Fig. 2c).

The Fenton reaction ability of Fe^2+^ ions can catalyze H_2_O_2_ to effectively produce cytotoxic ·OH species [35]. Methylene blue (MB), an ·OH indicator that can be degraded by ·OH, was chosen to investigate the ·OH production activity of BTFB@Fe@Van. As shown in Fig. 2d, the MB absorption by BTFB@Fe@Van at 664 nm significantly decreased when BTFB@Fe@Van was incubated in H_2_O_2_ under acidic conditions. A more noticeable reduction in MB absorption by BTFB@Fe@Van was observed after it was irradiated with a 1064 nm laser (1.0 W cm^−2^), indicating that NIR-II photothermal effect can accelerate the occurrence of the Fenton reaction. Moreover, electron spin resonance (ESR) spectra (Fig. 2e) showed that BTFB@Fe@Van could catalyze ·OH production in the presence of H_2_O_2_ at pH 5.5. Furthermore, with 1064 nm laser irradiation, the catalytic capability of BTFB@Fe@Van was significantly enhanced. In summary, these results confirmed that BTFB@Fe@Van exhibited catalytic activity for the Fenton reaction.

Due to the acidic microenvironment of the infection sites, the in vitro Van release performance of BTFB@Fe@Van was examined under different conditions. As shown in Fig. 2f, a low concentration of Van (less than 10%) was released after 4 h at a physiological pH of 7.4. By contrast, the release rate of Van was significantly fast, and its cumulative release reached 30% when BTFB@Fe@Van was kept in a solution with a pH of 5.5. The cumulative release of Van was further increased up to nearly 70% when the solution was irradiated with a 1064 nm laser (1.0 W cm^−2^). These results revealed that the phenylboronate ester bonds between BTF-PBA and Van in BTFB@Fe@Van endowed it with pH- and photothermal-triggered the release of Van at the infected site.

### In vitro antibacterial activity of BTFB@Fe@Van

**Fig. 3:**
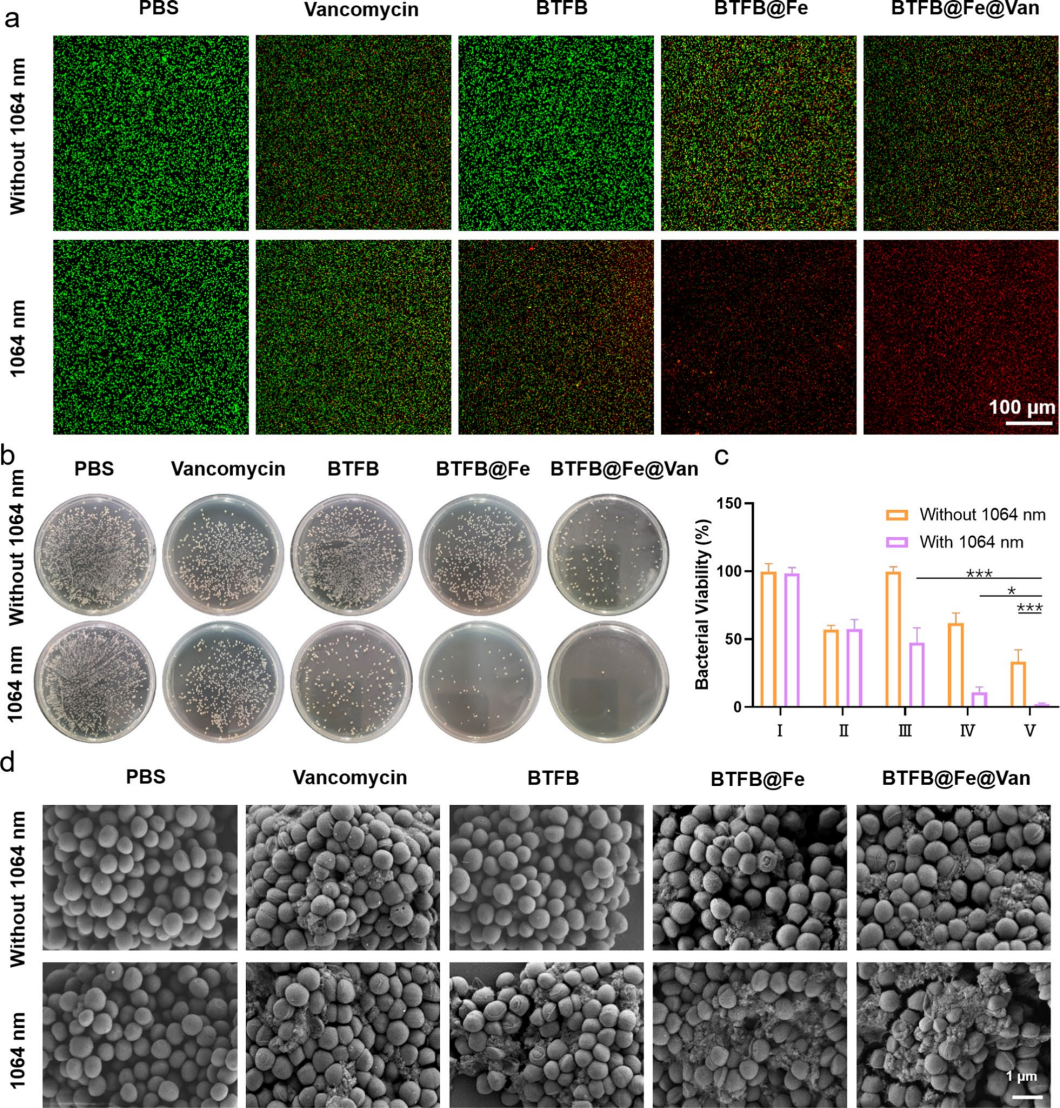
**a** Live/dead assay after different treatments. Scale bars: 100 μm. **b** Photographs of the LB agar plates of *S. aureus* after different treatments. **c** Bacterial viability of I: PBS, II: Van, III: BTFB, IV: BTFB@Fe, and V: BTFB@Fe@Van after different treatments. **d** SEM images of the bacteria after different treatments. Scale bars: 1 μm

The in vitro antibacterial efficacy of BTFB@Fe@Van was investigated using *S. aureus*, a major gram-positive bacterium associated with many acute and chronic infections encountered in clinical practice, as a model [36]. Encouraged by the satisfactory NIR-II photothermal capability and Fenton catalytic performance, as well as NIR-II PTT enhanced Van/Fe^2+^ release, we first examined intracellular ·OH generation using laser scanning confocal microscopy and employing a fluorescent probe (2′,7′-dichlorofluorescin diacetate) as the ·OH indicator. As shown in Additional file 1: Fig. S6, no detectable green fluorescence was observed in the PBS group. When BTFB@Fe and BTFB@Fe@Van were applied, bright green fluorescence was observed in the group, demonstrating elevated ·OH levels in *S. aureus*. The live/dead bacterial cell viability assay was performed to evaluate the in vitro antibacterial efficacy of BTFB@Fe@Van. Living and dead bacteria were stained with SYTO 9 and propidium iodide (PI; green fluorescence for living bacteria and red fluorescence for dead bacteria), respectively. The bacteria in the PBS, PBS + laser, and BTFB groups showed green fluorescence, indicating that all of the bacteria were alive (Fig. 3a). However, some red fluorescence appeared in the Van, BTFB + laser, BTFB@Fe, and BTFB@Fe@Van groups, which was due to the damage to *S. aureus* bacteria caused by Van, high temperature, and generated ·OH. By contrast, after treatment with BTFB@Fe@Van plus 1064 nm laser, almost all bacteria showed strong red fluorescence, indicating significant damage caused by the synergistic NIR-II PTT/CDT/antibiotic therapy applied using BTFB@Fe@Van.

The antibacterial effect of BTFB@Fe@Van on *S. aureus* was further evaluated using a Luria-Bertani (LB) agar plate assay (Fig. 3b). Numerous *S. aureus* colonies in the PBS and BTFB groups survived on the LB plates, revealing the inconspicuous bacteriostatic ability of BTFB. Subsequent to a quantitative analysis, the calculated bacterial survival rate of the Van group (57.4% ± 2.6%) was not significantly different from its value (57.7% ± 6.3%) obtained after exposing the group to 1064 nm laser, whereas the number of *S. aureus* colonies in BTFB + 1064 nm laser and BTFB@Fe groups tended to decrease with the corresponding bacterial survival rates standing at 47.4 ± 10.1% and 61.7 ± 7.05%, respectively, indicating diverse inhibitory effects on the bacterial growth due to hyperthermia and the presence of ·OH. The administration of two treatments demonstrated a therapeutic efficacy superior to that obtained using a single treatment (BTFB@Fe + 1064 nm: 10.8 ± 3.6%, BTFB@Fe@Van: 33.7 ± 7.8%). Importantly, the growth of colonies on plates treated with BTFB@Fe@Van plus 1064 nm laser was reduced and reached 1.9 ± 1.0%. The morphological changes in the bacteria subjected to different treatments were also studied using scanning electron microscopy (SEM, Fig. 3d). The PBS, PBS + laser, and BTFB groups retained intact and smooth membranes. A slight collapse and wrinkles were observed in the Van, Van + laser, and BTFB@Fe groups, whereas significant destruction was observed in the BTFB + laser, BTFB@Fe + laser, and BTFB@Fe@Van groups. Severe holes and wrinkles appeared on the surface of the bacteria in the BTFB@Fe@Van + 1064 nm laser group. These results demonstrated that BTFB@Fe@Van can effectively kill *S. aureus* through synergistic NIR-II PTT/CDT/antibiotic therapy.

### In vitro antibiofilm activity of BTFB@Fe@Van

**Fig. 4:**
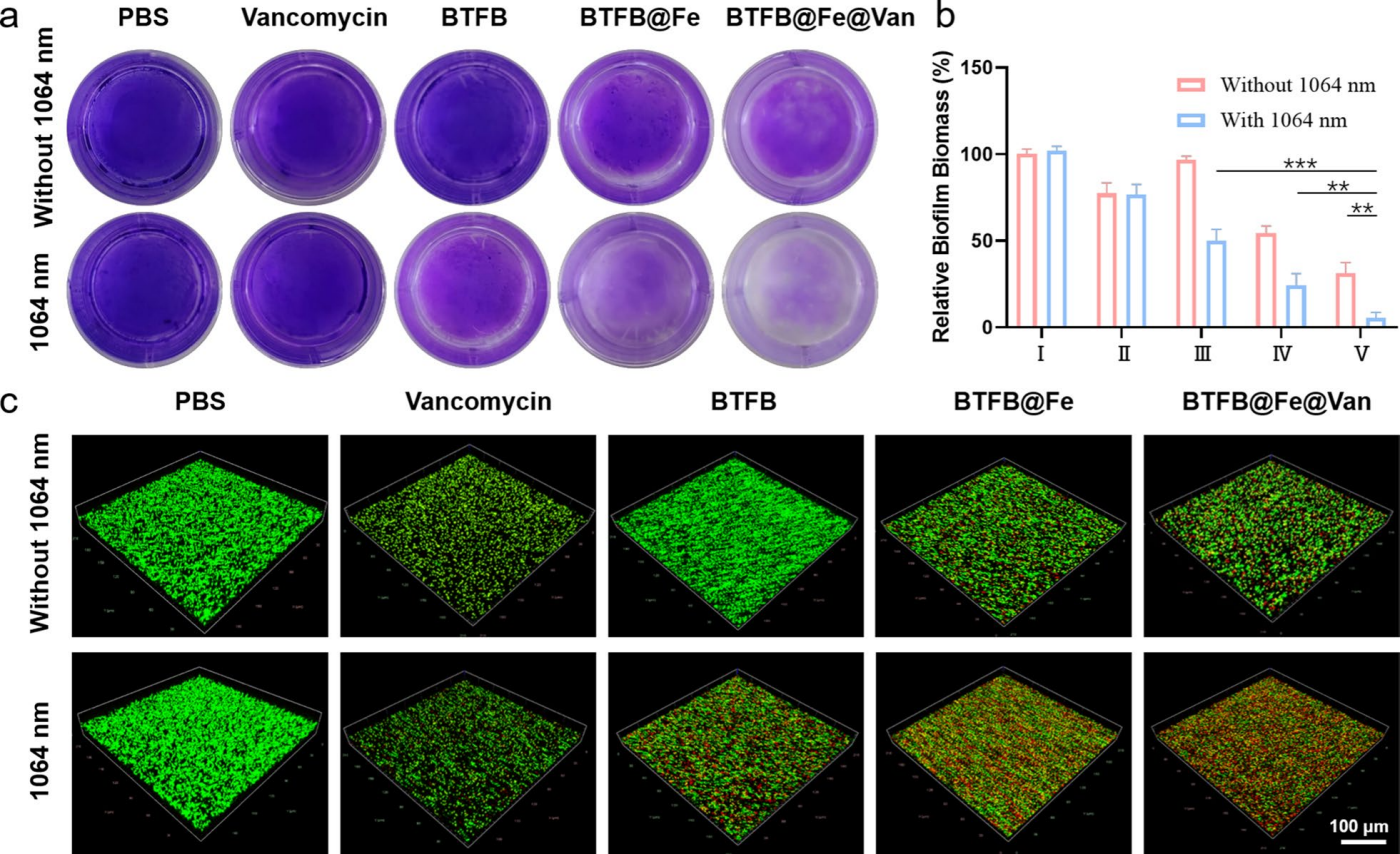
**a** Crystal violet staining of *S. aureus* biofilms after different treatments. **b** The corresponding quantified biomass of the biofilms of I: PBS, II: Van, III: BTFB, IV: BTFB@Fe, and V: BTFB@Fe@Van. **c** Three-dimensional CLSM images showing live/dead staining of *S. aureus* biofilms after different treatments (scale bars: 100 μm)

As bacteria colonize, they produce complicated and dense biofilms, and routine antibiotic therapy can hardly penetrate them. Moreover, bacterial biofilms offer strong resistance to host immune defenses and long-lasting antimicrobial resistance against infections [2, 3]. However, the emerging PTT and ROS triggered by BTFB@Fe@Van have great potential to destroy the membrane structures of bacterial biofilms and kill the bacteria, which will increase the efficacy of different antibiotics and combine them to combat bacteria. The antibiofilm ability of BTFB@Fe@Van was first assessed using crystal violet staining. As shown in Fig. 4a, b, the biofilms remained viable and structurally intact in the PBS, PBS + laser, and BTFB groups. By contrast, the biofilms in the Van, Van + laser, and BTFB@Fe groups lost biomass, while a more visible reduction was observed in the BTFB + laser, BTFB@Fe + laser, and BTFB@Fe@Van groups. The BTFB@Fe@Van + 1064 nm laser group produced excellent results in the biofilm inhibition test, completely disrupting the tight biofilms. Moreover, following various treatments, three-dimensional reconstruction of live/dead staining was performed via confocal laser scanning microscopy (CLSM) to determine antibacterial biofilm effects on them (Fig. 4c). As with crystal violet staining, different degrees of damage to the bacterial biofilms were observed when either single or multiple therapeutic methods were applied. The BTFB@Fe@Van + 1064 nm laser group greatly eradicated the mature biofilms and eliminated the bacteria, demonstrating a significant antibiofilm effect, which was due to the highly active synergistic effect of NIR-II PTT-enhanced CDT and antibiotic treatment.

### In vivo antibiofilm activity of BTFB@Fe@Van

**Fig. 5:**
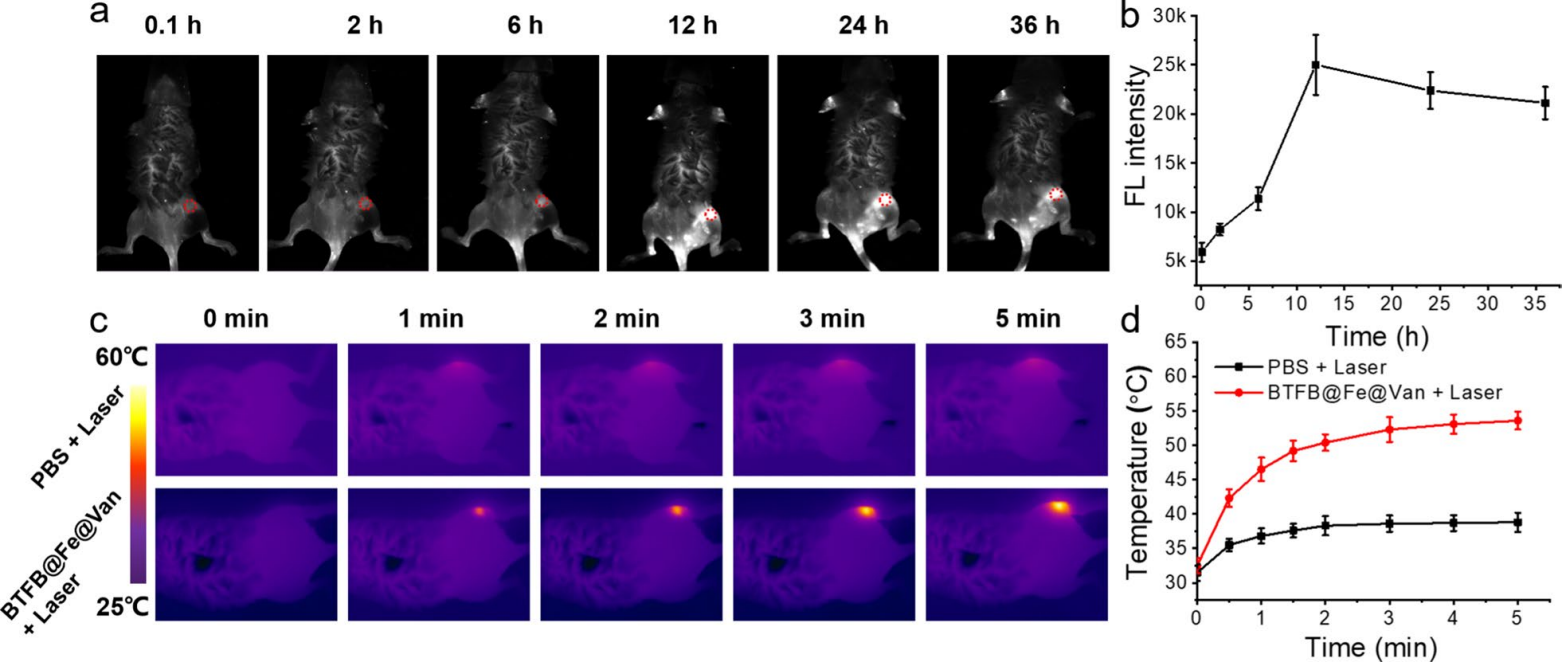
**a** NIR-II fluorescence images and **b** corresponding quantified NIR-II fluorescence signals of the infected mouse models at different time intervals. **c** Infrared photothermal images and **d** corresponding temperature variations at the infected sites with PBS or BTFB@Fe@Van treatment under 1064 nm laser irradiation (1.0 W cm^−2^, 5 min)

Encouraged by the in vitro synergistic therapeutic results, we investigated the in vivo synergic eradication of BTFB@Fe@Van in the presence of *S. aureus* biofilm infections. The model using severely infected mice was established to form the initial biofilm after 3 days of inoculation with *S. aureus* suspension. All infected wounds indicated obvious suppuration. First, BTFB@Fe @Van was intravenously injected into *S. aureus* biofilm-infected mice for NIR-II fluorescence imaging. As shown in Fig. 5a, the NIR-II fluorescence of BTFB@Fe@Van could be observed at the infection site 2 h after the injection and the fluorescence attained its maximum value at 12 h. Quantified NIR-II fluorescence signals of the infected mouse models are presented in Fig. 5b, which shows that the NIR-II fluorescence imaging signals at the peak time point were approximately five times stronger than those before the injection. The NIR-II photothermal temperature at the in vivo infection site was monitored using an infrared camera (Fig. 5c). The temperature at the infected site rapidly increased to 54 °C in mice treated with BTFB@Fe@Van after 5 min of irradiation (1064 nm, 1.0 W cm^−2^), whereas only a slight increase in the temperature (6 °C) was detected in the mice treated with PBS + 1064 nm laser irradiation (Fig. 5d), showing the great potential that PTT has in therapeutic applications involving living participants.

**Fig. 6:**
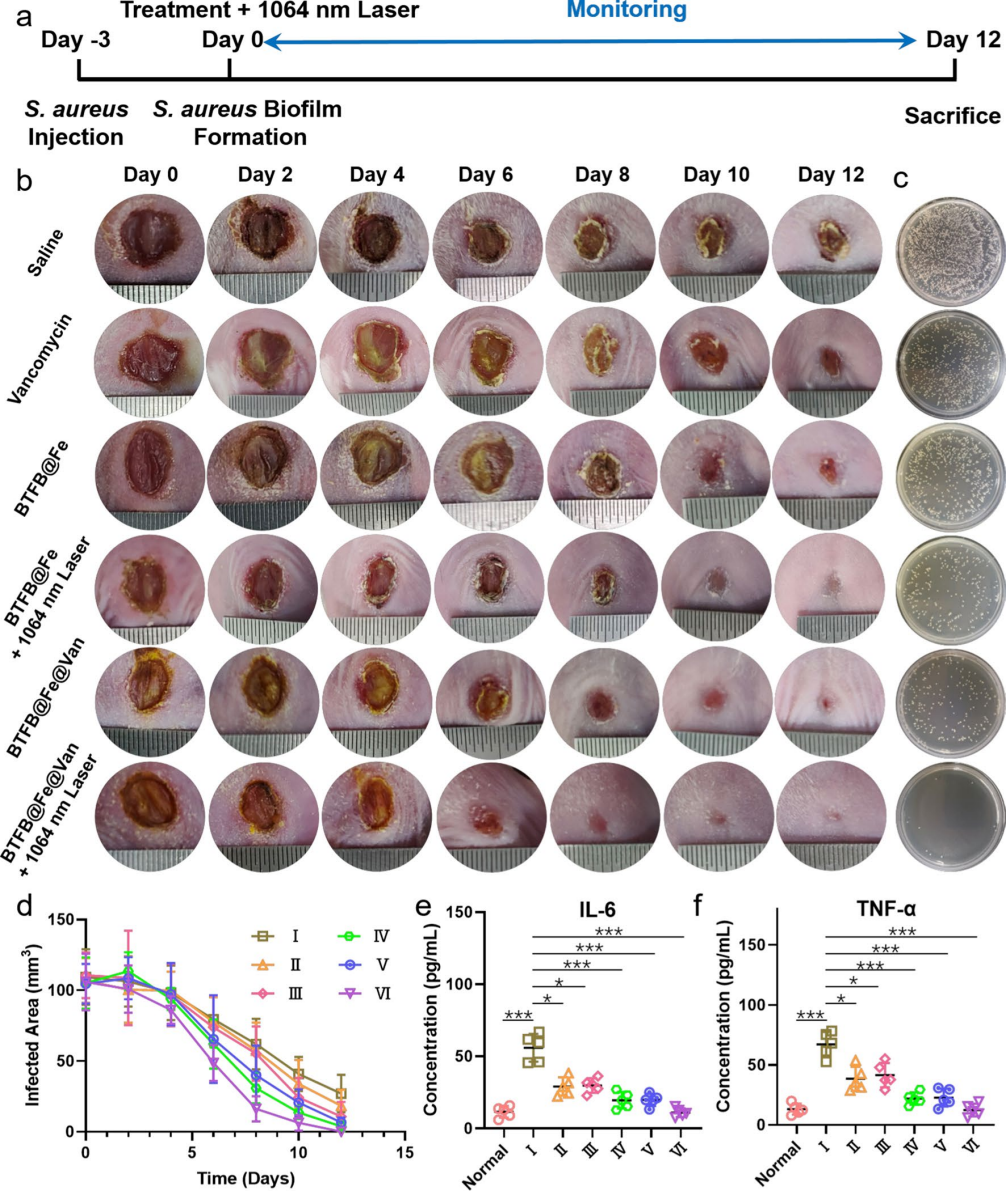
**a** Time line guidance for antibiofilm therapeutic process. **b** Digital photographs of the lesion site between 0 and 12 d after different treatments. **c** Photographs of the LB agar plates of *S. aureus* of the resected lesion site 12 d after the injection. **d** Quantitative analysis of the relative wound area throughout the therapeutic process. **e**, **f** Serum levels of cytokines IL-6 and TNF-α in I: Saline, II: Van, III: BTFB@Fe, IV: BTFB@Fe + 1064 nm laser, V: BTFB@Fe@Van, and VI: BTFB@Fe@Van + 1064 nm laser groups after different treatments

We randomly divided all *S. aureus* biofilm-infected mice into six groups: saline, Van, BTFB@Fe, BTFB@Fe + laser, BTFB@Fe@Van, and BTFB@Fe@Van + laser. We applied 1064 nm laser irradiation to the infected regions of the BTFB@Fe + and BTFB@Fe@Van + laser groups after administering the injection. We captured the macroscopic appearance of the wounds within 12 d after applying the different treatments, as shown in Fig. 6b. No significant effect was observed over time in the control saline group, its pus scabs became extremely thick. Meanwhile, the presence of subcutaneous wave motion in the group demonstrated pus accumulation, which was also confirmed by plate colony counting after the resection of infectious tissues (Fig. 6c). Unlike the control group, the Van, BTFB@Fe, BTFB@Fe + laser, and BTFB@Fe@Van groups showed low exudation and suppuration after being treated with either a single treatment or two combined treatments. The antibiofilm and wound healing performances of the BTFB@Fe@Van + laser group were superior to those of the other five groups. The infected site in the BTFB@Fe@Van + laser group appeared smooth without any pustules. The skin of the infected site started to gradually contract on the 6th day of treatment and the scar vanished on the 12th day of treatment, showing the most rapid recovery. Moreover, to further quantify the therapeutic effects of different treatments, the infected tissues were resected and the surviving bacterial numbers were counted at the end of the therapeutic process using plate colony counting. As shown in Fig. 6c, the number of surviving bacteria in the BTFB@Fe@Van + 1064 nm laser group was significantly lower than that in any of the other groups. The number of bacterial colonies was reduced by 98.19% compared to that in the control group. In addition, the wound size, which was quantitatively evaluated throughout the treatment period (Fig. 6d), exhibited a consistent trend. Moreover, we also investigated the serum level of the important inflammatory cytokines TNF-α and IL-6. Among the six groups, the BTFB@Fe@Van + 1064 nm laser group had the lowest TNF-α and IL-6 expression and showed no significant difference even compared to the normal mouse group (Fig. 6e, f). These results further demonstrated the excellent in vivo synergistic therapeutic performance of BTFB@Fe@Van upon 1064 nm laser irradiation.

**Fig. 7:**
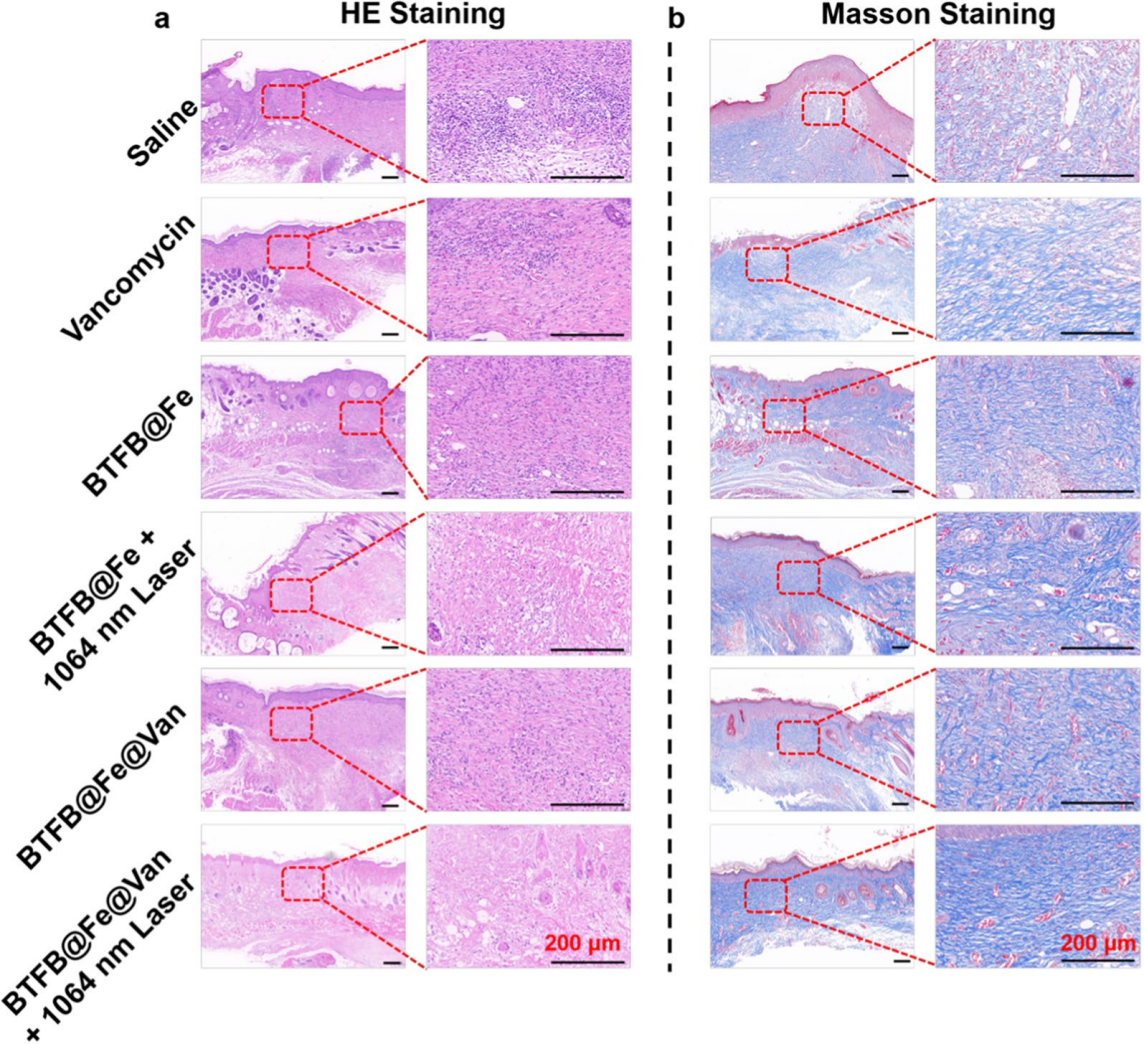
Representative **a** H&E staining and **b** Masson staining images of the wound subjected to different treatments. Scale bars: 200 μm

Furthermore, inflammation in the resected wounds was assessed using hematoxylin and eosin (H&E) staining (Fig. 7a) and Masson staining (Fig. 7b). The histological staining results showed a significant infiltration of inflammatory cells in the saline group, whereas the inflammatory responses in the BTFB@Fe + laser and BTFB@Fe@Van groups caused by bacterial colonization was less than that in the saline group because of the combined treatment it received. The BTFB@Fe@Van + 1064 nm laser group with its abundant collagen deposition and the low degree of neutrophil infiltration was superior to any other group, suggesting its favorable recovery ability. These results of in vivo *S. aureus* biofilm infection treatments collectively highlight the effectiveness of BTFB@Fe@Van in combating bacterial biofilm infections via the trimodal synergistic mechanism of NIR-II PTT, CDT, and antibiotics.

### Biocompatibility of BTFB@Fe@Van

**Fig. 8:**
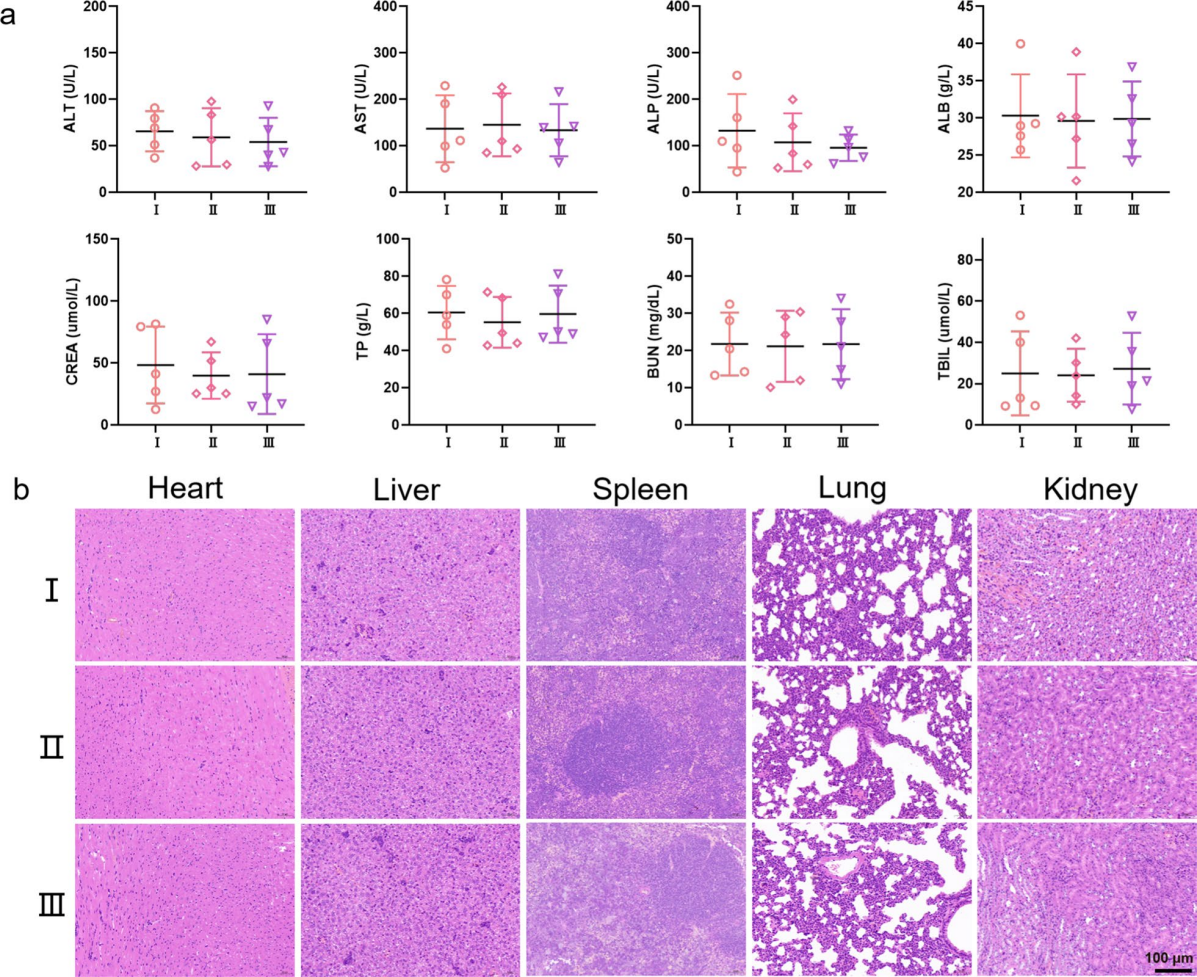
**a** Routine blood indexes of mice after different treatments at day 14 post-injection. **b** H&E staining effect of the main organ sections of the mice on I: Saline, II: BTFB@Fe, and III: BTFB@Fe@Van 14 d after the injection. Scale bar: 100 μm

In addition to verifying the antibiofilm properties of BTFB@Fe@Van, we conducted a series of experiments to assess its biocompatibility. The in vivo biocompatibility of BTFB@Fe@Van was further evaluated using blood samples to analyze liver and kidney functions. 14 d after intravenously injection, the biochemical index parameters of BTFB@Fe and BTFB@Fe@Van groups remained within the normal range and were not significantly different from those of the control group (Fig. 8a). Moreover, the histopathology images of the heart, liver, spleen, lung, and kidney of the BTFB@Fe and BTFB@Fe@Van groups further supported their good compatibility with no signs of infection or inflammation (Fig. 8b). These results demonstrated that nanoplatform BTFB@Fe@Van has a high in vivo biocompatibility, highlighting its potential for biomedical applications in living participants.

## Conclusions

In summary, a BME responsive nanoplatform (BTFB@Fe@Van) was developed to combat BBIs via a trimodal synergistic mechanism involving NIR-II PTT, CDT, and antibiotic. BTFB@Fe@Van produced bright NIR-II fluorescence images and excellent NIR-II PTT under 1064 nm laser irradiation. In addition, BTFB@Fe@Van could control the release of Van at the infection sites and catalyze the overexpression of H_2_O_2_ to initiate CDT. In vitro experiments showed that BTFB@Fe@Van possessed a noteworthy NIR-II PTT/CDT/antibiotic synergistic mechanism against BBIs. Moreover, BTFB@Fe@Van significantly promoted the recovery of the *S. aureus* biofilm-infected tissues and reduced the in vivo inflammatory response. Owing to the biological superiority of this trimodal synergistic strategy, BTFB@Fe@Van showed great potential as an intelligent nanoplatform for the eradication of drug-resistant BBIs.

### Supplementary Information

**Additional file 1.** Experimental section and additional figures associated with this article can be found in the online version.

## Data Availability

All data needed to support the conclusions are present in the paper and/or the Supporting Information. Additional data related to this study are available from the corresponding authors upon reasonable.
