# Supplementary material for: Near infrared II excitation nanoplatform for photothermal/chemodynamic/antibiotic synergistic therapy combating bacterial biofilm infections
Source: J Nanobiotechnology. 2023 Nov 24;21:446. doi: 10.1186/s12951-023-02212-7 (PMC10668414; doi:10.1186/s12951-023-02212-7)
Supplement: Supplementary file 1 — Additional file 1. Experimental section and additional figures associated with this article can be found in the online version. [file 12951_2023_2212_MOESM1_ESM.docx]

**Additional file 1**

Near Infrared II Excitation Nanoplatform for Photothermal/Chemodynamic/Antibiotic Synergistic Therapy Combating Bacterial Biofilm Infections

*Xuanzong Wang^1^,* *Chi Zhang^1^, Liuliang He^1^, Mingfei Li^1^, Pengfei Chen^2^, Wan Yang^2^, Pengfei Sun^2*^, Daifeng Li^1*^, Yi Zhang^1*^*

^1^Department of Orthopedics, The First Affiliated Hospital of Zhengzhou University, Zhengzhou, 450052, China.

^2^State Key Laboratory of Organic Electronics and Information Displays & Institute of Advanced Materials (IAM), Jiangsu Key Laboratory for Biosensors, Nanjing University of Posts & Telecommunications, Nanjing 210023, China.

*Correspondence:

Yi Zhang: zhangyi@zzu.edu.cn

Daifeng Li: lidaifeng@zzu.edu.cn

Pengfei Sun: iampfsun@njupt.edu.cn

***Reagents and Chemicals***

The NIR-II organic small molecule BTF-PBA (BTFB) was prepared based on our recent work [1]. DSPE-PEG2000 was purchased from Shanghai Yare Biotech, Inc. Luria-Bertani (LB) broth and agar powder were purchased from Solarbio Science & Technology Co., Ltd. (Shanghai, China). *Staphylococcus aureus* (*S.aureus* ATCC6538) was purchased from ATCC. Ethanol, Crystal Violet Ammonium Oxalate Solution (0.1%), Paraformaldehyde (4%), and Acetic Acid Solution (33%) were bought from Vazyme Biotech Co., Ltd. (Zhengzhou, China). Vancomycin (Van) Hydrochloride was prepared by Sinopharm Chemical Reagents Co., Ltd. (Shanghai, China). The LIVE/DEAD BacLight Bacterial Viability Kit (L7012) was obtained from Thermo Fisher Scientific Co., Ltd. The usage of the reagents and chemicals mentioned above were without further processing or purification.

***Characterization***

The morphology of nanoparticles was determined by transmission electron microscope (HT7700, TEM) under 100 KV acceleration voltage. Dynamic light scattering (DLS) analysis using a commercial laser light scattering spectrometer (ALV-7004; ALV, Langan, Germany) was equipped with a multi-τ digital time correlator and a He-Ne laser (at λ = 632.8 nm). The ⟨*D*_h_⟩ data was extracted by CONTIN analysis. All samples we used for testing were optically cleared with a Millipore filter (0.45 μm). Tests were performed at room temperature at a scattering Angle of 90°. NIR-II fluorescence spectra were measured using an NIR-II spectrophotometer (Fluorolog 3, Horiba). NIR InGaAs was selected as the detector, with an excitation wavelength of 1064 nm obtained from a diode laser operating at 25.0 ± 0.5 °C. After the raw emission data were collected, the fluorescence signal was further confirmed and corrected for the sensitivity of InGaAs detector profile and output through the T1c channel. The laser was purchased from Changchun New Industries Optoelectronics Technology Co., Ltd. The *in vitro* and *in vivo* NIR-II FI experiments were conducted on an NIR-II imaging system (Wuhan Grand-imaging Technology Co., Ltd) with 1064 nm LP filters under the 808 nm laser irradiation. A 640 × 512 pixel two-dimensional InGaAs array from Princeton Instruments in NIR-II fluorescence windows was equipped in this NIR-II imaging system. All photothermal tests were detected using a Fotric 225 instrument (IR thermal camera, ± 2 °C) purchased from Fotric. (Shanghai, China).

***Preparation of BTFB NPs***

First, dissolve 1.0 mg BTFB in 1.0 mL MeOH solution. Then, the BTFB solution (1.0 mL, 1.0 mg/mL) was quickly injected into the DSPE-mPEG_2000_ (15 mg) aqueous solution (10 mL), and ultrasonic treatment was conducted for 3 min, and the organic solvent was removed by dialysis.

***Preparation of BTFB@Fe NPs***

The freshly prepared ferrous sulfate solution (0.1 M, 0.5 mL) was put into the prefabricated BTFB NPs (1.0 mL) and vigorously stirred overnight. The preparation method was referred to BTFB@Fe NPs.

***Preparation of BTFB@Van NPs***

Dissolving 1.0 mg BTFB and 1.0 mg Van in 1.0 mL MeOH solution. Then, the mixed solution was quickly injected into DSPE-PEG_2000_ (15 mg) aqueous solution (10 mL), treated with ultrasound for 3 min, and the organic solvent was removed by dialysis.

***Preparation of BTFB@Fe@Van NPs***

The freshly prepared ferrous sulfate solution (0.1 M, 0.5 mL) was put into the prefabricated BTFB@Van (1.0 mL) and vigorously stirred overnight. The preparation method was referred to BTFB@Fe@Van.

***The loading capacity of Vancomycin in BTFB@Fe@Van NPs***

In order to evaluate the loading capacity of Van in BTFB@Fe@Van NPs, several different concentrations of water solution of Van were firstly prepared and detected by UV spectrophotometer to get the absorption intensities at 280 nm, and then make a standard curve with them as shown in Figure S5. Then, BTFB NPs and BTFB@Fe@Van NPs of the same concentration were prepared respectively. The absorbance of BTFB NPs and BTFB@Fe@Van NPs at 280 nm was determined, and Van capacity was calculated by standard curve according to Beer-Lambert law. The carrying capacity calculated by UV method was about 28 %.

***In vitro Photothermal Effect and Photothermal Conversion Efficiency***

To evaluate the photothermal effects of BTFB@Fe@Van NPs, 1064 nm laser (1.0 W/cm^2^, 5 min) was used to irradiate 200 μL nanoparticle solutions with concentrations of 25, 50, 100 and 200 μg/mL, respectively. The temperature changes of BTFB@Fe@Van NPs solution were recorded by infrared thermography, and these data were recorded every 30 s. To study the photothermal conversion (ŋ) behavior of BTFB@Fe@Van NPs, a thermal imaging camera (Fotric 225, Fotric Precision Instruments, USA, ± 2 °C) was used to perform the thermal imaging of NPs in an aqueous solution. First, an aqueous solution of nanoparticles (0.1 mg/mL) was configured, 200 μL of which was added into a 250 μL centrifuge tube. The temperature changes of a fixed concentration of BTFB@Fe@Van NPs (0.1 mg/mL) were irradiated with a 1064 nm laser (1.0 W/cm^2^, 5 min), and then the laser was shut off. Finally, we could obtain a temperature increase and drop curve.

The photothermal conversion efficiency (ŋ) was calculated using equations (1) and (2) expressed below. The photothermal conversion efficiency of the BTFB@Fe@Van NPs was determined through the collected data and equation.

ŋ = [hS(T_max_-T_suur_)-Q_dis_]/[I(1-10^-A1064^)] (1)

τ_s_ =m_D_C_D_/hS (2)

The parameters S, h, Tmax, Tsurr, Qdis, I and A1064 are the container’s surface area, heat-transfer coefficient, maximum laser-trigger temperature, indoor temperature, heat dissipation caused by the light absorbing of quartz cuvette, intensity of laser (1.0 W/cm^2^) and absorbance of BTFB@Fe@Van NPs at 1064 nm, respectively.

Parameter τs is the time constant of the sample system. The parameters m_D_ and C_D_ are the mass and heat capacity of the solvent, respectively.

***In vitro Vancomycin Release***

*In vitro* drug release profile of Van was determined by loading 1.0 mL of BTFB@Fe@Van NPs samples into dialysis tubing (MW, 50000), which was submerged into 10 mL of phosphate-buffered saline (pH = 7.4 and 5.5) as dissolution media. The release experiments were performed with and without 1.0 W cm^-2^ 1064 nm laser irradiation for 5 min at initial time of experiment at 37 ℃. The mixture was then incubated in water bath at 37 ℃ under continuous shaking. At predetermined time points, the daily sate was taken out to estimate the amount of drug released, while the same amount of fresh PBS was added back and kept in a shaker for further study. The Van concentration in the samples was measured by absorption spectroscopy.

***In Vitro ·OH Detection***

Firstly, aqueous solutions with different pH values (pH = 7.4 and 5.5) were prepared, each solution (150 μL) was added to the 96-well plate, and H_2_O_2_ (50 μL, 0.5 mM) and MB (50 μL, 1 mM) were added to each well, and the mixed solution was used as the test environment. Then, BTFB@Fe@Van (0.1 mg/mL, 100 μL) was added to each well. Finally, the absorbance of each well was measured by PowerWaveXS/XS2 microplate spectrophotometer at 664 nm at room temperature, and ·OH formation were quantified by the decrease of MB absorbance at 664 nm.

***Bacteria Culture***

*S.aureus* (ATCC 6538) was cultured in the sterilized LB broth agar medium at 37℃, 150 rpm overnight. After two generations of propagation, the bacteria reached an exponential growth rate. Then, the concentration of the bacteria was diluted to 10^7^ CFU/mL with the absorbance of the bacterial suspension at 600 nm (OD_600_ = 0.1).

***In Vitro Antibacterial Assay***

With the bacteria of a concentration 10^7^ CFU/mL prepared above, the bacterial suspension was divided into 10 groups randomly which accepted corresponding measures: (1) PBS, (2) PBS + 1064 nm laser, (3) Van, (4) Van + 1064 nm laser, (5) BTFB, (6) BTFB + 1064 nm laser, (7) BTFB@Fe, (8) BTFB@Fe + 1064 nm laser, (9) BTFB@Fe@Van, (10) BTFB@Fe@Van + 1064 nm laser. After 12 h co-culture of the chemicals and bacterial suspension at 37℃, 60 rpm overnight, group (2), (4), (6), (8), (10) received 1064 nm laser irradiation (1.0 W/cm^2^, 5 mins) separately. Afterwards, all of the 10 groups suspension were diluted 5000 times and extracted 100 uL of the diluted suspension to spread on the LB agar plate. After 16 h incubation, the bacterial colonies of the 10 groups were counted accordingly. Bacterial viability was measured by the following formula.

$$Bacterial Viability=\frac{bacterial colonies in sample}{bacterial colonies in PBS}\times100\%$$

***Live / Dead Bacterial Staining Assay***

The 10 groups bacterial of suspensions were centrifuged at 4000 rpm for 10 mins. After discarding the supernatant and washing with PBS, the residua were dispersed in normal saline and then stained with the mix of SYTO 9 and PI (LIVE/DEAD BacLight Bacterial Viability Kit) for 30 mins, 37℃ in the incubator. Soon after washing with PBS, each group was redispersed in 100 uL normal saline for use. Then each sample was extracted 10 uL to spread on the object slide to be observed by Zeiss LSM 980 confocal microscope.

***Scanning Electron Microscopy***

After *S.aureus* was resuscitated and propagated for two generations, the bacteria incubated with the corresponding drugs overnight, then followed by 1064 nm laser irradiation. After irradiation, the medium was discarded and the bacteria were washed with PBS for three times, and then fixed with 2.5% glutaraldehyde fixative for 30 mins. After fixation, the specimens were washed with PBS for three times and fixed with 1% OsO_4_ in dark for 2 h. Afterwards, different concentrations of ethanol and isoamyl acetate were added successively to dehydrate, and then the specimens were dried by Critical Point Dryer (Quorum K850). After drying, the specimens were sputter-coated with gold for 30 s using Lon Sputtering Apparatus (HITACHI MC1000) and then observed by Scanning Electron Microscope (HITACHI Regulus 8100).

***Crystal Violet Assay***

Firstly, the concentration of *S.aureus* suspension was adjusted to 10^7^ CFU/mL and subsequently inoculated in the 24-well plate for 3 days. After receiving different treatments, the wells were washed with PBS, fixed with Paraformaldehyde (4%) and stained with crystal violet (0.1%). Then the stained biofilms were observed on the microscope. Finally, the stained biofilms were dissolved with the acetic acid solution (33%) and the absorbance of the solution was measured at 590 nm with a microplate reader (Multiskan GO).

***Biofilm Morphology via Confocal Laser Scanning Microscopy***

Firstly, the density of *S.aureus* was diluted to 10^7^ CFU/mL (OD_600_ = 0.1) to cultivate biofilm in the 24-well plate. After 3 days of cultivation at 37℃, the integrated biofilm was developed on the coverslip in the 24-well plate. Later, diverse treatments were applied according to the same measures as mentioned above. Subsequently after staining with SYTO 9 and PI, the biofilms were assessed under the CLSM (Zeiss LSM 980) to analyze the morphology of the biofilms.

***Animal Studies***

The animal experiments were authorized by the Institutional Committee on the Ethics of Animal Experiments of Zhengzhou University (Zhengzhou, China). All of the animal studies complied with the Guide for the Care and Use of Laboratory Animals of the National Institutes of Health. The Balb/C mice (8 weeks old, female, 18-20 g) were provided by Beijing Vital River Laboratory Animal Technology Co., Ltd. For the establishment of biofilm-infected wound mouse model, round skin defects with a diameter of 6 mm were constructed on the posterior dorsal skin of mice using a mouse skin tissue punch sampler. Then, a concentration of 10^8^ CFU/mL *S.aureus* suspension was prepared to spread on the round skin defects of the mice. 3 d after the infection modeling, the dense biofilm was formed and the mice were divided into 6 groups (Saline, Van, BTFB@Fe, BTFB@Fe + 1064 nm laser, BTFB@Fe@Van, BTFB@Fe@Van + 1064 nm laser, 1064 nm laser irradiation: 1.0 W/cm^2^, 5 mins). During the whole animal experiment process, the weight of the mice, the diameters of wound infection and the images of the infection model were recorded once every two days. After 12 d of treatments, all the mice were sacrificed. Then, the infected tissues dissected from the mice accepted two measures: (1) homogenized in normal saline for bacteria colony calculation. (2) fixed in paraformaldehyde (4%), embedded in paraffin wax and sliced for HE staining and Masson staining. At the same time, the blood samples were collected from the mice to detect the serum TNF-α and IL-1β levels by means of flow cytometry.

***Biocompatibility***

The normal Balb/C mice (8 weeks old, female, 18-20 g) were divided into 3 groups (Saline, BTFB@Fe, and BTFB@Fe@Van) to test the biocompatibility. 14 days after intravenous injection of BTFB@Fe and BTFB@Fe@Van, the mice were sacrificed and the blood was collected via eyeball extirpating to monitor the other relevant serum biochemical indexes through microplate reader. Moreover, the important organs (heart, liver, spleen, lung and kidney) were also resected and excised to fix in paraformaldehyde (4%) for further HE staining. In the last step, all the tissue slices were observed with Olympus IX 70 inverted microscope.

***Flow Cytometry***

The levels of IL-6 and TNF-α cytokines in blood samples were detected by Cytokines Detection Kit through flow cytometry. Firstly, the standard substance in the kit and the mice plasma samples were diluted. 50 µL of the diluted standard substance and 50 µL of diluted plasma samples were added to the 96-well plate, and then the two parts were mixed in a vortex. After that, the 96-well plate was placed on the magnetic plate, in the shaker and the 37℃ incubators successively. Also, 200 µL of washing liquor, 100 µL of antibody detection and 100 µL of streptavitin labeled by phycoglobin were added in turn. Finally, 200 µL of washing liquor was added to each well of the 96-well plate to resuspend the samples and all the samples were detected by flow cytometer.

***In vivo NIR-II Fluorescence Imaging***

The mice were imaged alive by anesthetizing them with isoflurane during the test time. The real-time *in vivo* NIR-II fluorescence imaging was performed at diﬀerent post-injection times by using an *in vivo* NIR-II fluorescence imaging system with 1064 nm LP filters. The excitation wavelength was 808 nm produced by a semiconductor laser. The analysis of the signal intensity of NIR-II image was performed using the NIR-II *in vivo* imaging system software.

***Statistical Analysis***

All experiments were carried out in at least triplicate, and the results are shown as the mean ± standard deviation (S.D.). Unless otherwise stated, statistical comparisons between various groups were performed using with one-way analysis of variance (ANOVA) with a corrected *p* value below 0.05 considered statistically to be significant (**p* < 0.05, ***p* < 0.01, ****p* < 0.001).


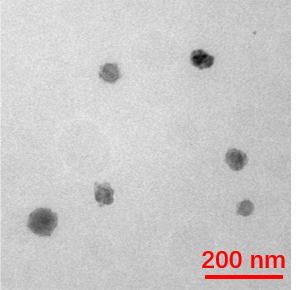


**Figure S1.** Transmission electron microscope image of BTFB.


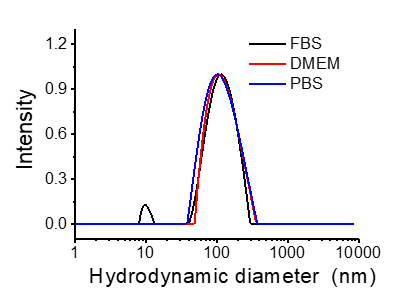


**Figure S2.** Particle size of BTFB@Fe@Van in FBS, PBS and DMEM.


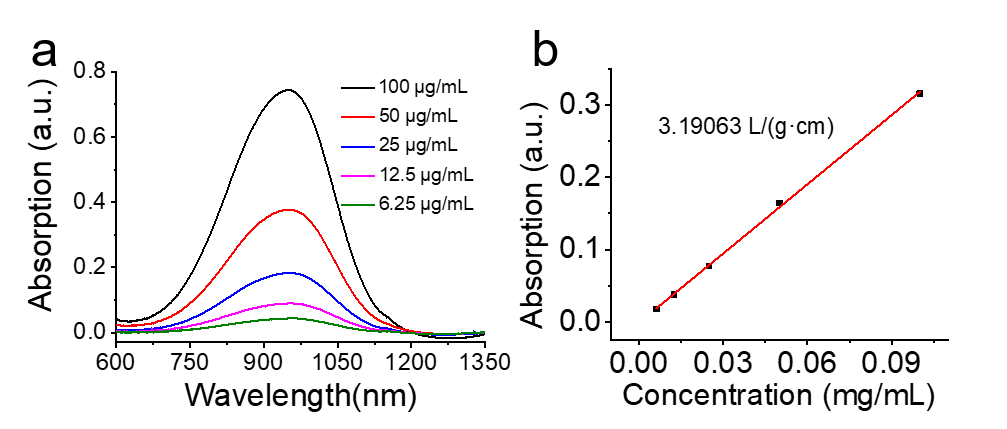


**Figure S3.** (a) Absorption spectra of BTFB@Fe@Van at different concentrations. (b) Extinction coefficient of BTFB@Fe@Van at 1064 nm.


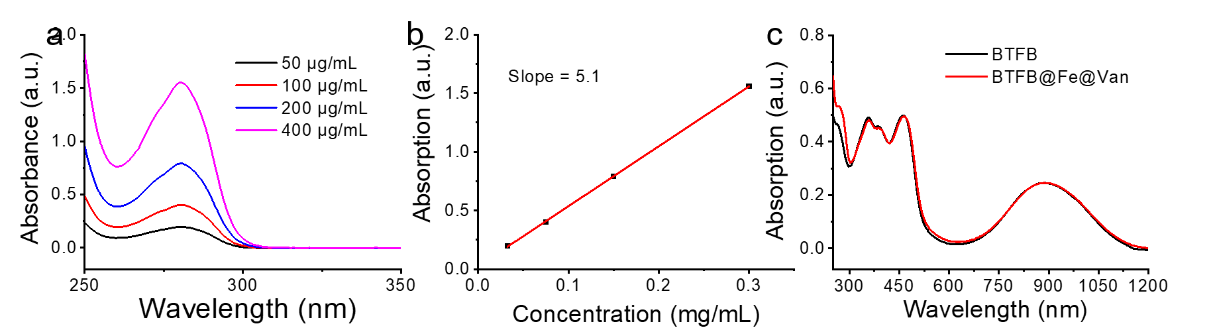


**Figure S4.** (a) Absorption spectra of vancomycin at different concentrations in water. (b) Standard curve of vancomycin at 280 nm. (c) Absorption spectra of BTFB@Fe@Van and BTFB in water.


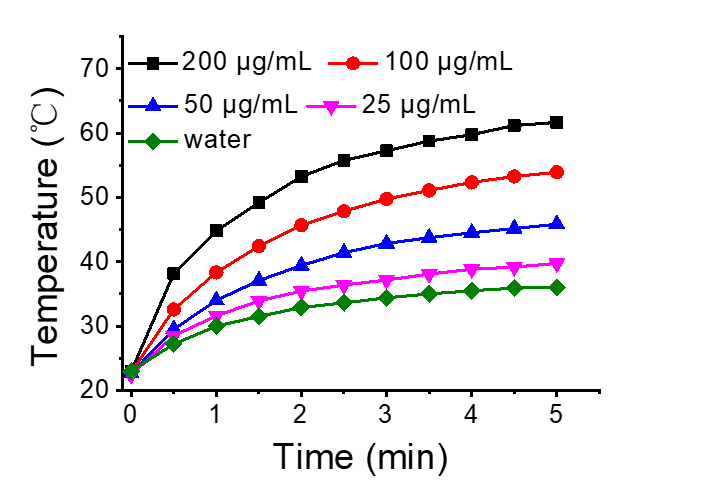


**Figure S5.** Photothermal curves of different concentrations of BTFB@Fe@Van and water.


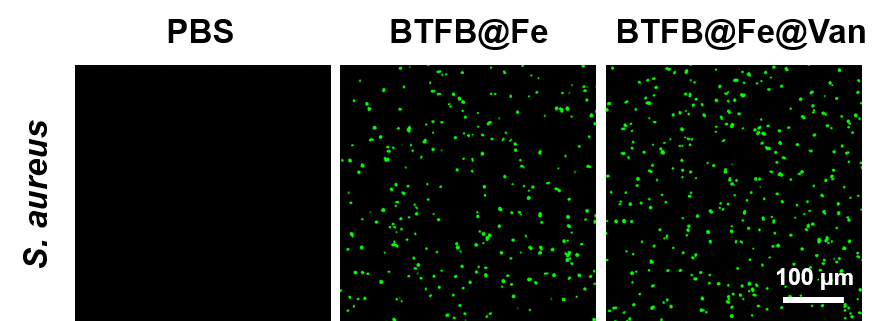


**Figure S6.** Intracellular ·OH levels after different treatments. Scale bar: 100 μm.

**References**

[1] P. Sun, W. Yang, J. He, L. He, P. Chen, W. Xu, Q. Shen, D. Li, Q. Fan, *Advanced Healthcare Materials*, 2023, 2302099, DOI: 10.1002/adhm.202302099.
